# Combined T1-mapping and tissue tracking analysis predicts severity of ischemic injury following acute STEMI—an Oxford Acute Myocardial Infarction (OxAMI) study

**DOI:** 10.1007/s10554-019-01542-8

**Published:** 2019-02-16

**Authors:** Malgorzata Wamil, Alessandra Borlotti, Dan Liu, André Briosa e Gala, Alessia Bracco, Mohammad Alkhalil, Giovanni Luigi De Maria, Stefan K. Piechnik, Vanessa M. Ferreira, Adrian P. Banning, Rajesh K. Kharbanda, Stefan Neubauer, Robin P. Choudhury, Keith M. Channon, Erica Dall’Armellina

**Affiliations:** 1 Division of Cardiovascular Medicine, Radcliffe Department of Medicine, John Radcliffe Hospital, University of Oxford, Oxford, OX3 9DU UK; 2 NIHR Oxford Biomedical Research Centre, John Radcliffe Hospital, Oxford, OX3 9DU UK; 3 Acute Vascular Imaging Centre, Radcliffe Department of Medicine, John Radcliffe Hospital, Oxford, OX3 9DU UK; 4 The Leeds Institute of Cardiovascular and Metabolic Medicine, Division of Biomedical Imaging, University of Leeds, Leeds, UK

**Keywords:** Strain, Myocardial infarction, Cardiac magnetic resonance

## Abstract

Early risk stratification after ST-segment–elevation myocardial infarction (STEMI) is of major clinical importance. Strain quantifies myocardial deformation and can demonstrate abnormal global and segmental myocardial function in acute ischaemia. Native T1-mapping allows assessment of the severity of acute ischemic injury, however its clinical applicability early post MI is limited by the complex dynamic changes happening in the myocardium post MI. We aimed to explore relationship between T1-mapping and feature tracking imaging, to establish whether combined analysis of these parameters could predict recovery after STEMI. 96 STEMI patients (aged 60 ± 11) prospectively recruited in the Oxford Acute Myocardial Infarction (OxAMI) study underwent 3T-CMR scans acutely (within 53 ± 32 h from primary percutaneous coronary intervention) and at 6 months (6M). The imaging protocol included: cine, ShMOLLI T1-mapping and late gadolinium enhancement (LGE). Segments were divided in the infarct, adjacent and remote zones based on the presence of LGE. Peak circumferential (Ecc) and radial (Err) strain was assessed using cvi42 software. Acute segmental strain correlated with segmental T1-mapping values (T1 vs. Err − 0.75 ± 0.25, p < 0.01; T1 vs. Ecc 0.72 ± 0.32, p < 0.01) and with LGE segmental injury (LGE vs. Err − 0.56 ± 0.29, p < 0.01; LGE vs. Ecc 0.54 ± 0.35, p < 0.01). Moreover, acute segmental T1 and strain predicted segmental LGE transmurality on 6M scans (p < 0.001, r = 0.5). Multiple regression analysis confirmed combined analysis of global Ecc and T1-mapping was significantly better than either method alone in predicting final infarct size at 6M (r = 0.556 vs r = 0.473 for global T1 only and r = 0.476 for global Ecc only, p < 0.001). This novel CMR method combining T1-mapping and feature tracking analysis of acute CMR scans predicts LGE transmurality and infarct size at 6M following STEMI.

## Introduction

Myocardial infarct (MI) size is a major determinant of the left ventricular (LV) dysfunction and long term remodelling in patients post ST-segment–elevation myocardial infarction (STEMI) [1]. Although primary percutaneous coronary intervention (PPCI) revolutionised the management of acute MI patients by allowing early intervention and saving myocardium from irreversible injury, there is still considerable and partially unexplained variability in the long-term outcome [2]. Therefore, accurate early risk stratification is of major clinical importance. Despite the broad use of LV ejection fraction (EF) in assessing post-MI LV remodelling, its prognostic value is poor [3]. Standard cardiovascular magnetic resonance (CMR) tissue characterization techniques such as late gadolinium enhancement (LGE) and T2Weighted (T2W) imaging provide several markers of long-term prognosis and remodelling [4]. Despite the availability of several highly accurate imaging markers (such as infarct size, transmurality, salvage myocardium), there is yet no definite consensus on the most relevant parameter. Additionally, novel CMR findings arising from imaging in the first hours post ischemia [5], seem to suggest the unfolding of complex dynamic tissue changes [6, 7] hampering the prognostic relevance of standard LGE techniques [8, 9] and indicating the need for more accurate techniques for stratification of patients early after MI.

By allowing quantitative assessment of the severity of the ischemic injury on a continuous scale, CMR mapping techniques allow accurate characterisation of the composition and viability of the myocardium after MI [10–12] providing additional predictive markers of remodelling and mortality in the infarcted core [13], and remote myocardium [14–16]. The early longitudinal changes happening in the myocardium, limit the ability to define the clinical significance of the severity of the myocardial injury earlier than 72 h. A marker defining the functional impact of such changes and its effects on late remodelling is needed. Post ischemia, the changes happening in the tissue composition lead to impairment in myocardial deformation [17]. Historically, tagging was used to assess myocardial deformation by CMR, however, the applicability of such a technique in acute MI patients is challenging due to long scan times, breath holds and lengthy post processing [18]. Another recently validated CMR method, feature tracking imaging (FTI), allows measurements of circumferential, radial and longitudinal myocardial strain by tracking tissue voxel motion in cine CMR images avoiding the acquisition of additional sequences [19–22]. Whilst the incremental prognostic value of FTI in MI patients has been recently demonstrated above EF and infarct size [23], the relationship between myocardial strain and markers of severity of ischemic injury, as assessed by native quantitative mapping techniques, has not yet been fully defined. Here, we sought to investigate the additional predictive value of clinical outcome for markers of acute myocardial deformation such as circumferential (Ecc) and radial strain (Err) by FTI, to T1-mapping tissue characterization in STEMI patients.

## Methods

### Patient population

96 STEMI patients who underwent PPCI were prospectively enrolled in the study at the John Radcliffe Hospital as part of the OxAMI Study. This was a pre-specified study within the OxAMI research programme. Patients were eligible if the onset of symptoms had been within 12 h before PPCI and if they had ST-segment elevation of at least 0.1 mV in ≥ 2 contiguous limb leads or at least 0.2 mV ≥ 2 contiguous precordial leads. Patients with previous MI, previous revascularization procedure (coronary artery bypass grafts or PCI), severe heart valve disease, known cardiomyopathy, or hemodynamic instability lasting ≥ 12 h after revascularization were excluded. Further exclusion criteria were contraindications to CMR, including implanted pacemakers, defibrillators, or other metallic implanted devices and claustrophobia. Acute clinical management was at the discretion of the responsible physician, with the intention to reflect contemporary practice and guidelines. The study protocol was approved by the local ethics committee, and all patients gave written informed consent.

### Cardiac magnetic resonance

Patients underwent 3 T CMR scan (either MAGNETOM TIM Trio or MAGNETOM Verio, Siemens Healthcare, Erlangen, Germany) at 2 time points: acutely (within 53 ± 32 h post PPCI) and at 6 months (6M). Matching short axis slices covering the LV were acquired using an established CMR protocol including: cine, native T1-mapping using the Shortened Modified Look-Locker Inversion recovery (ShMOLLI), and LGE as described in the Table 1 [24]. ShMOLLI T1 maps were generated from 5 to 7 SSFP images with variable inversion preparation time as described previously [24, 25]. Briefly, typical acquisition parameters were: TE/TR = 1.07/2.14 ms, flip angle = 35°, FOV = 340 × 255 mm, matrix size = 192 × 144, 107 phase encoding steps, actual experimental voxel size = 1.8 × 1.8 × 8 mm, interpolated reconstructed voxel size = 0.9 × 0.9 × 8 mm, GRAPPA = 2, 24 reference lines, cardiac delay time TD = S2 500 ms and 206 ms acquisition time for single image, phase partial Fourier 6/8. LGE was performed with a T1-weighted segmented inversion recovery gradient echo-phase sensitive-inversion recovery (GRE_PSIR) sequence (TE/TR = 2.5 ms/5 ms, voxel size 1.8 × 1.4 × 8 mm, flip angle 20°). LGE were collected 10–15 min after the administration of 0.1 mmol/kg contrast agent (Gadodiamide, Omniscan TM, GE Healthcare, Amersham, UK). The inversion time was adjusted for optimal nulling of remote normal myocardium. SSFP cine images were acquired using retrospective gating (TE/TR = 1.4/3.2 ms; flip angle = 50°; pixel size: 1.6 × 1.6 mm). Two to three-fold accelerated parallel imaging (GRAPPA) was used to shorten the breath-hold on LGE imaging.

**Table 1:** Characteristics of CMR sequences

Imaging method	Cine	ShMOLLI T1 maps	LGE
Sequence	SSFP	5–7 SSFP images	T1-weighted GRE_PSIR
TR, ms	3.2	2.14	5
TE, ms	1.4	1.07	2.5
Flip angle	50°	35°	20°
In-plane resolution	1.6 × 1.6	1.8 × 1.8	1.8 × 1.4
Slice thickness, mm	8	8	8
Other parameters			Optimal T1 to null the remote myocardium

GRE_PSIR gradient echo-phase sensitive-inversion recovery sequence; SSFP steady-state free precession sequence

### CMR imaging analysis

#### Global analysis

Anonymised images were analysed using cvi42 software (Circle Cardiovascular Imaging Inc., Calgary, Canada) by two experienced operators (MW and AB; both > 5 years of CMR analysis experience) blinded to CMR measurements of the same patient across two time points and included LV volumes, quantification of LV% oedema on T1 maps [26], LV% necrosis/scar on LGE, global, circumferential and radial strain as previously described [22, 27]. The LGE signal intensity threshold for quantification of necrosis/scar was set at 5 standard deviations (SD) above the reference ROI in the remote unaffected myocardium [26, 28]. Global mean T1-mapping values were calculated averaging segmental T1-mapping values per patient. The final infarct size was defined as the LV% LGE at 6M.

#### Segmental analysis

**Fig. 1:**
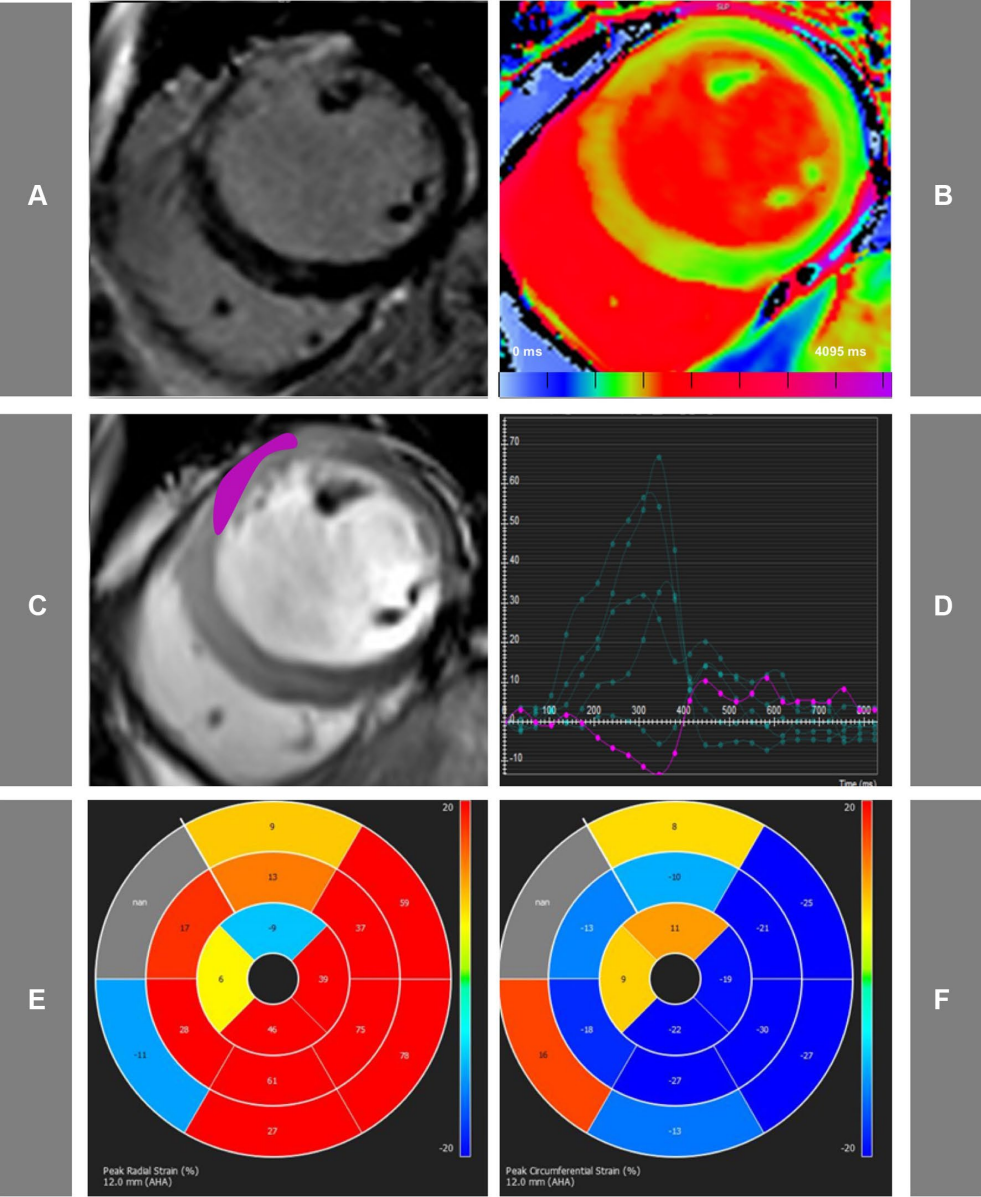
Representative acute CMR images used for the assessment of STEMI patients. Matching mid ventricular short axis slices acquired using LGE PSIR images (**a**), native T1-mapping (**b**), functional cine imaging (**c**). **d** The cvi42-derived tissue tracking analysis, whilst **e** and **f** display representative bull’s eye maps for radial and circumferential strain assessment, respectively. In this case of acute anterior myocardial infarction, the myocardium co-localized with the enhanced areas on LGE images, show prolonged T1 values and abnormal peak radial (Err) and circumferential (Ecc) strain

Segmental analysis was performed on the short-axis (SA) images (Fig. 1). For all the techniques SA images were divided in six equiangular segments with the anterior RV–LV junction as reference point. The following segmental measurements were assessed: LGE percentage fraction, peak radial (Err) and circumferential (Ecc) strain (%), native T1 values. Segmental LGE was calculated as percentage fraction of the segment volume. Segmental peak radial (Err) and circumferential (Ecc) strain (%) were analysed based on the previously published methods [29] and were calculated using 50 cords at end-systole and end-diastole using the same, standard epicardial and endocardial contours as drawn for diastolic and systolic volume analysis [30]. Endo- and epicardial contours were drawn manually in all slices by one skilled observer. T1 maps underwent strict and extensive quality control as previously described [24]. Segmental T1 values were derived from short-axis T1 maps using in-house dedicated software MC-ROI (Interactive Data Language, version 6.1, Exelis Visual Information Solutions, Boulder, Colorado). Segments with MVO were excluded from the analysis. Far apical slices with partial volume effects and slices with visible outflow tracts were excluded from the study.

**Fig. 2:**
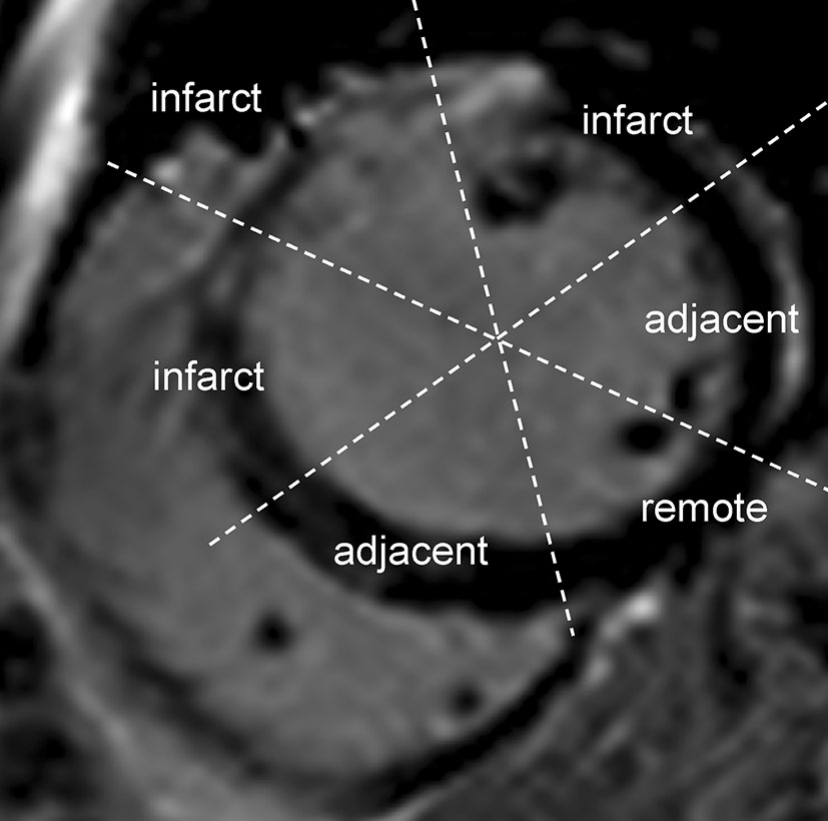
Definition of the infarcted and the adjacent myocardium. This is a representative short axis image of a patient with anterior myocardial infarction by late gadolinium enhancement. Segments with LGE > 25% were identified as infarcted, whilst segments located on the same plane contiguously to the infarcted segments and with LGE < 25% were defined as adjacent; myocardium with no LGE and 180° from the infarct was defined as remote

Cvi42 software was used to assess LGE transmurality (Tissue characterisation: LGE module). Briefly, contours were drawn manually around LV endo- and epicardium. The LGE signal intensity threshold for quantification of necrosis was set at five standard deviations (SD) above the reference ROI in the remote unaffected myocardium [26, 28]. Myocardium was divided into six standard segments and LGE transmurality was reported per segment. Segments were graded according to peak LGE transmurality in end-diastole as follows (1) > 0 to ≤ 25% (2) > 25 to ≤ 50% (3) > 50 to ≤ 75% and (4) > 75%. Segments showing LGE > 25% were defined as infarcted, whilst segments with no LGE and located 180 degrees from the infarct, were defined as remote, as previously described [31]. Segments located on the same plane contiguously to the infarcted segments and with LGE < 25% were defined as adjacent (Fig. 2). Infarcted segments with persistent LGE ≥ 50% at 6M scans were identified as irreversibly damaged. Segments within the infarct zone with LGE ≥ 25% on the acute scan and negative LGE on 6M scan were identified as recovered segments.

### Statistical analysis

Statistical analyses were performed using IBM SPSS Statistics (version 22.0, IBM Corporation, Armonk, NY). Continuous data are expressed as mean ± SD except where otherwise specified. Categorical variable was expressed as n%. The normality of the data was assessed using the Shapiro–Wilk test or the Kolmogorov–Smirnov test depending on the size of samples tested. Those variables that did not follow a normal distribution are presented as medians with interquartile range (median (IQR)). Comparisons between groups were performed with t-test or one-way ANOVA where normality was met or with the non-parametric Kruskal–Wallis H. Test when data normality was violated (differences in median T1 values, LGE, Err and Ecc in infarct, adjacent and remote segments). Correlation was expressed as Pearson or Spearman correlation coefficient. To account for the fact that segmental T1 values and segmental peak strain analysis could not be treated as independent samples, the following approach was utilised: correlation coefficient values from the segmental analysis of two imaging biomarkers was first derived for each patient, then transformed with the Fisher’s z’ transformation that converts Pearson’s r’s to the normally distributed variable z and subsequently analysed by t-test [32]. CASTS (combined analysis of segmental native T1-mapping and segmental tissue tracking analysis) is a term, which we used for the combined acute segmental strain and acute T1-mapping analysis and was derived from logistic regression and then tested as predictive probability against strain and T1-mapping values separately. The linear regression was used to assess the performance of the combined native T1-mapping and strain analysis in predicting the final scar size at 6M time. Multiple regression analysis was used to test the contribution of T1 mapping, Ecc and Err in predicting 6M peak LGE transmurality. P-values less than 0.05 were considered statistically significant.

## Results

Patient characteristics are given in Table 2. One hundred and twenty patients were recruited of which 24 were excluded due to the following reasons: claustrophobia (n = 10), scanner failure (n = 2) and poor CMR image quality (due to breathing artefacts on acute T1 maps or LGE images) (n = 12). Out of the remaining 96 STEMI patients with acute CMR (53 ± 36 h post PCI), 59 patients underwent a 6M follow up scan. Reasons for not returning for follow-up CMR scans were as follows: withdrawal from the study (n = 30), death (n = 3), and implantable cardioverter-defibrillator (n = 4).

**Table 2:** Baseline characteristics of the study population

	Mean ± SD
Age, year	60 ± 11
Sex, M/F	68/28
Risk factors, n (%)	
Diabetes	10 (10)
Smoker	26 (27)
Hypertension	33 (34)
Hyperlipidaemia	34 (35)
Family history of CHD	35 (36)
Target vessel n (%)	
LAD	43 (45)
LCx	7 (7)
RCA	26 (27)
Peak troponin (mg/l)	206 ± 290
Pain to Balloon time (mins)	242 ± 180
PPCI to CMR time (h)	53 ± 36
No. of vessels diseased, n (%)	
1	72 (75)
2	17 (19)
3	7 (6)
TIMI flow pre PCI, n (%)	
0	66 (80)
1	5 (6)
2	10 (12)
3	1 (1)
TIMI flow post PCI, n (%)	
1	1 (1)
2	4 (5)
3	68 (93)

LAD indicates left anterior descending artery, LCx left circumflex artery, RCA right coronary artery

### CMR findings

**Fig. 3:**
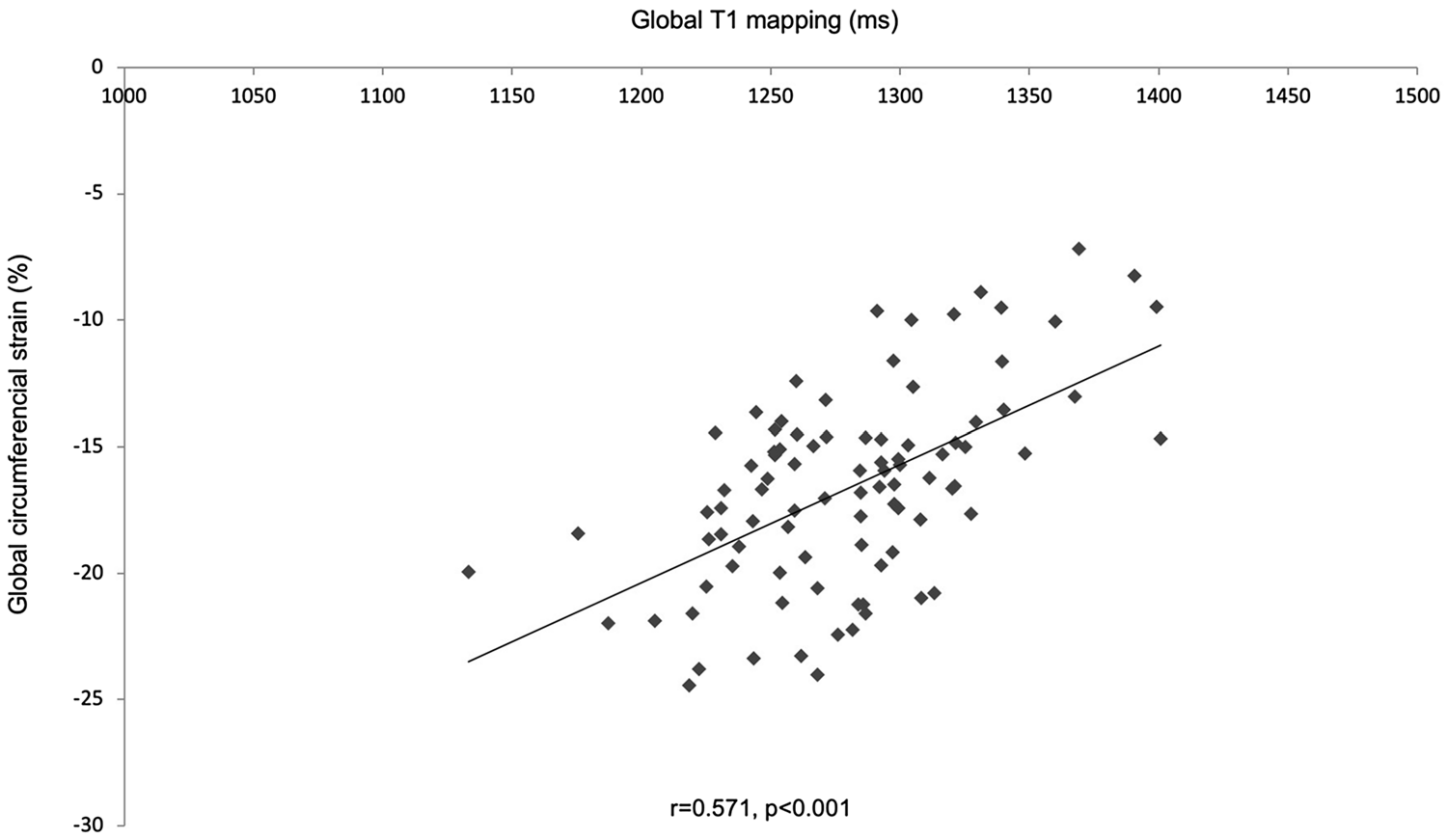
Correlation between global native T1-mapping and global circumferential strain. Global analysis of averaged T1 values (calculated as averaged values per patient) and global circumferential strain analysis (derived from tissue tracking analysis cvi42 software) showed a significant correlation (r = 0.571, p < 0.001)

CMR findings are summarized in Table 3. On LGE images, only one patient had no evidence of enhancement. At 6M, EF improved by 5% (p < 0.001) (Table 3). There was a significant correlation between global strain and global native T1-mapping: T1 versus Err r = − 0.495, p < 0.001, T1 versus Ecc r = − 0.571, p < 0.001 (Fig. 3).

**Table 3:** CMR findings

	Acute mean ± SD (n = 96)	6M mean ± SD (n = 59)	*p*-Value
EF (%)	46 ± 8	51 ± 9	< 0.001
EDV (ml)	164 ± 43	163 ± 39	0.9
ESV (ml)	89 ± 33	78 ± 31	< 0.01
SV (ml)	73 ± 20	86 ± 18	< 0.01
Oedema by T1-mapping, (%LV)	42 ± 14		
LGE, (%LV)	24 ± 14	16 ± 10	< 0.001
Myocardial salvage index [(Oedema%LV-%LV LGE)/Oedema %LV]	47 ± 22		
MVO (%LV)	2 ± 3		
Global LV T1 (ms)	1280 ± 47	1214 ± 75	< 0.001
Global circumferential strain (%)	− 16.6 ± 3.8		
Global radial strain (%)	29.6 ± 9		

*EF* ejection fraction, *EDV* end-diastolic volume, *ESV* end-systolic volume, *LGE* late gadolinium enhancement, *LV* left ventricle, *MVO* microvascular obstruction

### Segmental analysis

**Fig. 4:**
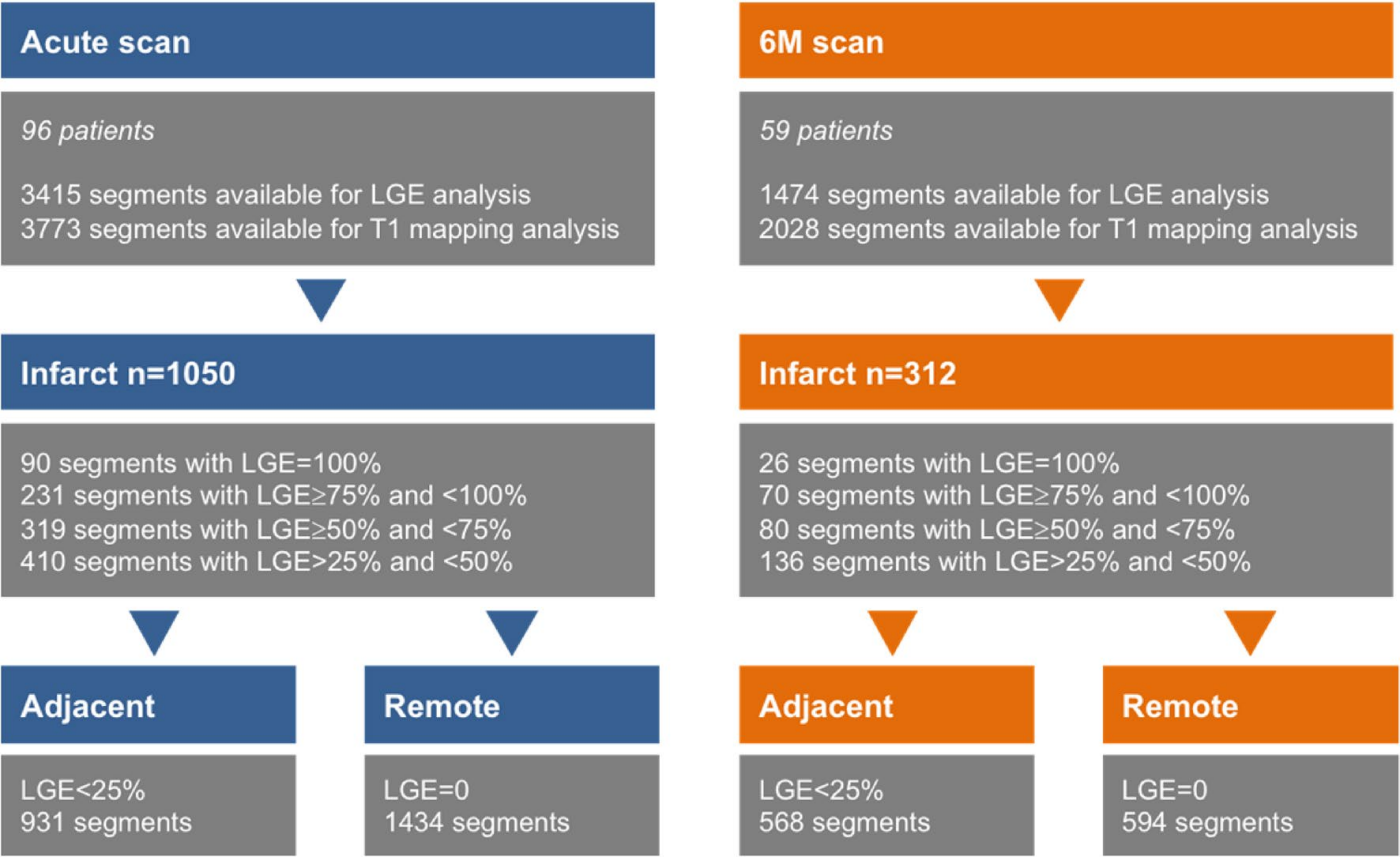
The figure shows the distribution of segments in the study on the acute and 6M scans according to LGE transmurality. Segments were divided into infarct, adjacent and remote zones based on LGE transmurality and their location as described in the “Methods”

A total of 3415 acute segments and 1474 on the 6M scans were suitable for analysis. Out of all acute segments, there were 90 segments with LGE = 100%, 231 segments with LGE ≥ 75% and < 100%, 319 segments with LGE ≥ 50% and < 75% and 410 segments with LGE > 25% and < 50% (Fig. 4). As shown in Table 4, acute native T1 values in infarcted and adjacent segments were significantly prolonged compared to remote myocardium (1357 ± 89 ms infarcted vs. 1245 ± 100 ms adjacent vs. 1215 ± 87 ms, remote, p < 0.001). Acutely, 20% of the remote segments (293/1434) showed significantly prolonged T1 values comparing to normal values [33] (1229 ± 76 ms vs. 1180 ± 36 ms, p < 0.001). Native T1 values recovered significantly over time in infarcted, adjacent myocardium and also in those remote segments showing prolongation of T1 acutely (infarct: acute scan 1357 ± 89 ms vs. 6M scan 1235 ± 99 ms, p < 0.01; adjacent zones: acute scan 1245 ± 100 ms vs. 6M scan 1193 ± 66, ms p < 0.01; remote acute scan: 1215 ± 87 ms vs. 6M scan 1185 ± 66 ms, p < 0.01).

**Table 4:** CMR segmental values of strain and tissue injury acutely and at 6M

		Infarct	Adjacent	Remote	*p* Value	*p* Value
		n = 1050	n = 931	n = 1434	(infarct vs. remote)	(adjacent vs. remote)
Acute CMR	T1 (ms)	1357 ± 89	1245 ± 100	1215 ± 87	< 0.001	< 0.001
	LGE (%)	52 ± 53	0 ± 6	0 ± 0	< 0.001	< 0.001
	Err (%)	12 (13)	27 (19)	35 (23)	< 0.001	< 0.01
	Ecc (%)	− 10 (9)	− 17 (8)	− 20 (9)	< 0.001	0.07
6M CMR	T1 (ms)	1235 ± 99	1193 ± 66	1185 ± 66	< 0.001	0.387
	LGE (%)	36 (56)	0 (5)	0 (0)	< 0.001	< 0.001

Data are expressed as mean ± SD or median (IQR)

### Relationship between severity of ischemic injury and strain

Both Err and Ecc worsened progressively as acute T1 values increased from remote to adjacent to infarcted myocardium (median (IQR) Err_infarct_ = 12 (13)% vs. Err_remote_ = 35 (23)%, p < 0.01; Ecc_infarct_ = − 10 (9)% vs. Ecc_remote_ = − 20 (9)%, p < 0.01) (Table 5). The difference between Ecc in adjacent versus remote segments did not reach significance (Ecc_adjacent_ = − 20 (8)% vs. Ecc_remote_ zone − 21 (7)%; p = 0.07). Err and Ecc values varied significantly between segments grouped by LGE transmurality (Table 5). On a patient by patient basis, myocardial deformation by Err and Ecc correlated significantly with T1 values (T1 vs. Err r = − 0.75 ± 0.25, p < 0.01; T1 vs. Ecc r = 0.72 ± 0.32, p < 0.01) and with LGE segmental fraction (LGE vs. Err r = − 0.56 ± 0.29, p < 0.01; LGE vs. Ecc r = 0.54 ± 0.35, p < 0.01) (Table 6).

**Table 5:** CMR segmental T1, Err and Ecc values according to LGE transmurality

		LGE < 25%	LGE 25–50%	LGE 50–75%	LGE ≥ 75%	*p* Value
		n = 2538	n = 319	n = 225	n = 333	(difference between groups)
Acute CMR	T1 (ms)	1229 ± 94	1332 ± 86	1374 ± 91	1378 ± 84	< 0.001
	Err (%)	31.4 (25)	14 (15)	11 (10)	8.8 (9)	< 0.001
	Ecc (%)	− 19 (10)	− 12 (10)	− 10 (8)	− 7.5 (7)	< 0.001
6M CMR	T1 (ms)	1197 ± 68	1210 ± 83	1245 ± 96	1268 ± 87	< 0.001
*p* Value (acute vs. 6M T1)		< 0.001	< 0.001	< 0.001	< 0.001	

Data are expressed as mean ± SD or median (IQR)

**Table 6:** Correlation coefficients between acute segmental native T1-mapping values and segmental radial (Err) and circumferential (Ecc) strain as well as LGE

	T1 vs. Err	T1 vs. Ecc	LGE vs. Err	LGE vs. Ecc
Mean ± SD	− 0.75 ± 0.25	0.72 ± 0.32	− 0.56 ± 0.29	0.54 ± 0.35
p Value	p < 0.001	p < 0.001	p < 0.001	p < 0.001
n Patients	96	96	96	96
Segments	3773	3773	3773	3773

All segments were first analysed for correlations per patient n = 96 and then these correlation coefficients were compared with one-sample t-test

### The role of normal circumferential strain in the assessment of functional recovery of the adjacent segments

Table 4 shows significantly prolonged native T1 values in the adjacent segments comparing to the infarct and remote zones. A trend towards a statistically significant difference in circumferential strain between adjacent and remote zones was shown (adjacent segments Ecc − 20 ± 8% vs. remote zone − 21 ± 7%, p = 0.07). There was no difference in the native T1 values in the remote and adjacent segments on the 6M scan suggesting a functional recovery of these segments.

### The additional value of combined acute segmental strain and T1-mapping (CASTS) for 6M prediction of LGE peak segmental transmurality

In order to assess whether acute myocardial deformation in addition to myocardial composition, as assessed by T1 values, predict the 6M peak LGE segmental transmurality, we performed a multiple regression analysis including acute segmental T1, Err and Ecc values as independent variables. When combined these variables significantly predicted 6M segmental LGE transmurality (p < 0.001, r = 0.5). Furthermore, we analysed the contribution of each of the independent variables to the prediction of the 6M peak LGE segmental transmurality. When T1 and Ecc values were used together both variables significantly improved the prediction of final segmental LGE transmurality (p < 0.001 for each of them), however, Err did not add to the prediction of 6M segmental LGE transmurality (Err p = 0.662). Thus, in further analyses we used T1-mapping combined with Ecc only.

### Predictive value of the combined analysis of global strain and T1-mapping in the assessment of 6M infarct size

In view of the fact, that global rather than segmental stain has been previously shown to predict cardiac remodelling after STEMI [34], we wanted to examine the performance of global values obtained for the tissue tracking analysis and global T1 values (calculated as averaged values per patient). We then compared the combined predictive value of the global analysis of circumferential strain and global T1 values. We confirmed that combined analysis of these two biomarkers was a good predictor of the final infarct scar on the follow-up scan at 6M (p < 0.001, r = 0.556) and that both biomarkers when assessed globally improved significantly the prediction of the scar size (as assessed per patient as total LGE in %; standardized coefficients for global T1 values 0.323, p = 0.018, for global Ecc 0.329, p = 0.016). Furthermore, we showed that combined analysis of these two global biomarkers was significantly outperforming global T1-mapping or global circumferential strain analysis alone (r = 0.556 vs. r = 0.473 for T1 only and r = 0.476 for Ecc only; p < 0.001).

## Discussion

**Fig. 5:**
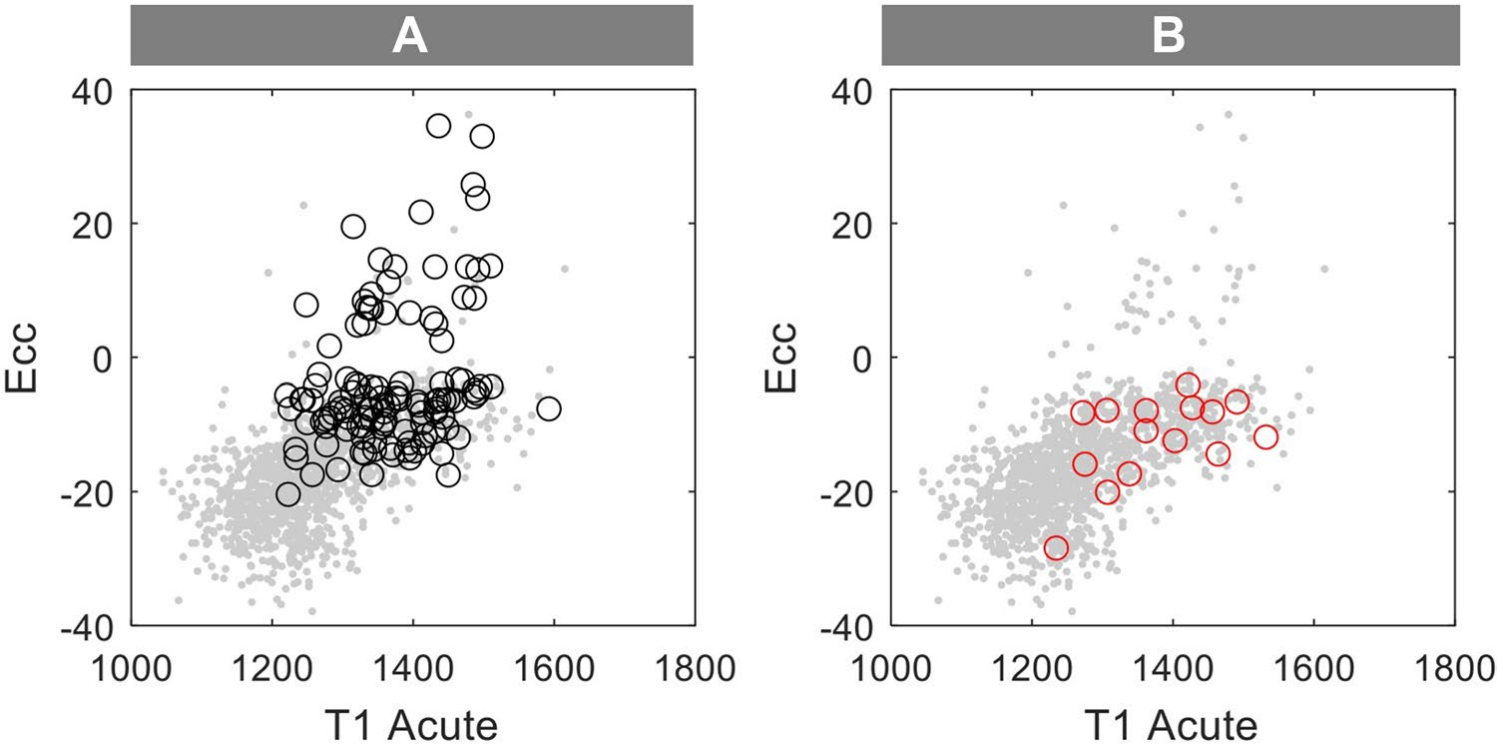
Performance of the combined analysis of acute T1-mapping and circumferential strain (CASTS) for functional assessment post-MI. Figure represents the performance of segmental native T1-mapping combined with segmental Ecc in identifying **a** irreversibly damaged segments visualised as black circles (> 50% LGE on the acute and 6M scan, segments with MVO were excluded from the analysis) and **b** normalised segments visualised as red circles (LGE = 0 on the 6M scan and LGE ≥ 25% on the acute scan and in the area of infarction). Grey dots represent all assessed segments by these two methods

In patients with acute MI, novel CMR imaging markers derived from either tissue characterisations mapping techniques or FTI methods have been shown to have incremental prognostic value compared to standard LGE infarct size and EF [13, 15, 23]. By determining voxel wise T1 values on a continuous scale as measurements of the tissue composition, T1-mapping provides additional information on the severity of injury allowing distinguishing between reversible and irreversible injury. The dynamic changes happening in the tissue composition during the healing phase post MI [35] could result in variations in the acute T1-mapping values [7]. Hence, there is a demonstrable need for an additional stable imaging parameter capable of increasing the accuracy of early risk stratification. Regional strain might be well suited to increase further the predictive value of mapping techniques by reflecting abnormal segmental myocardial deformation as a result of acute changes in tissue composition. By investigating the interrelationship between native T1-mapping and regional strain in acute MI, our study shows the additional value of the combined acute segmental strain and T1-mapping analysis (CASTS) in predicting irreversibility of the ischemic injury compared to either of these imaging markers alone (Fig. 5).

Whilst peak segmental strain and native T1-mapping analysis reliably identifies infarct and remote zones, in areas adjacent to the ischemic myocardium, circumferential strain measures did not significantly differ from remote myocardium despite of the high T1 values. This suggests that native T1-mapping and peak circumferential strain analysis combined is able to distinguish between infarcted, irreversibly damaged segments and peri-infarct zones that have a high chance to recover.

Moreover, we observed significant prolongation of the native T1 values in 20% of the non-ischaemic zones and their recovery on the follow-up scans. Significantly, all these segments had normal values of circumferential strain. This observation is particularly potent in light of gadolinium-free T1-mapping techniques potentially replacing contrast methods altogether. In our study, segments were divided in the infarct, adjacent and remote zones based on the presence of LGE. Thus, if one would rely exclusively on the analysis of segments of the native T1 values, these remote segments with increased T1-mapping values might get wrongly labeled as infarcted. Conversely, the addition of circumferential strain allows us to accurately identify these segments. This is particularly true when assessing zones adjacent to the infarct. Similar observations that were previously reported by Carrick et al. [14] and by Reinstadler et al. [15], who also showed increased native T1-mapping values in the non-ischaemic myocardium post-MI. The authors confirmed that the remote native T1-mapping was independently predictive of MACE at 6 months after STEMI. They postulated these alterations in the non-ischaemic zones T1 values were a result of inflammation and hypercellularity. Pre-clinical studies strongly support the presence of pro-inflammatory macrophages in the non-ischaemic remote myocardium in MI models [36]. Thus, we postulate that an increase in the remote zones T1 values acutely post-MI could reflect an exacerbated tissue inflammation affecting the non-ischaemic myocardium (remote and adjacent zones) early post-MI and that circumferential strain can help to distinguish these segments with high inflammatory changes but full chances to recover from those that were irreversibly damaged.

## Limitations

The additive value of the combined analysis of segmental native T1 mapping and tissue tracking is somewhat smaller than anticipated. This could be explained by the fact that actually both imaging biomarkers measure the same process, namely acute injury caused by inflammation and oedema of the myocardium. Thus, changes in the tissue characteristic will influence changes in the tissue deformation measured by the peak strain. This observation is supported by our findings that show significant correlation between the percentage of global oedema and global strain.

Another important limitation of our study is a significant drop out in the follow-up CMR scans. 59 out of 96 recruited subjects returned for the 6M follow up scans. We decided to include all recruited patients in our study but we acknowledge the analysis of the predictive value of the combined analysis of global strain and T1-mapping in the assessment of 6M infarct size was performed on those subjects, which underwent full study protocol including 6M scans.

## Conclusions

This novel CMR method combining T1-mapping and strain analysis of acute CMR scans improves the prediction of irreversible ischaemic injury following STEMI. Further studies using alternative mapping modalities and investigating the longitudinal changes over the early hours post MI, will be needed to establish a combined protocol of native T1 mapping and feature tracking assessment as an alternative to the standard LGE method, limiting the CMR protocol to non-contrast acquisitions.

## Abbreviations

MI: Myocardial infarction
STEMI: ST-segment elevation myocardial infarction
PPCI: Primary percutaneous intervention
LGE: Late gadolinium enhancement
CASTS: The combined acute segmental native T1-mapping and the strain analysis
Ecc: Circumferential strain
Err: Radial strain
EF: Ejection fraction
T2W: T2Weighted images
ECV: Myocardial extracellular volume
FTI: Feature tracking imaging
GRE_PSIR: T1-weighted segmented inversion recovery gradient echo-phase sensitive-inversion recovery
SSFP: Steady state free precession
